# Epigenetic age acceleration and mortality risk prediction in US adults

**DOI:** 10.1007/s11357-025-01604-x

**Published:** 2025-03-17

**Authors:** Angelico Mendy, Tesfaye B. Mersha

**Affiliations:** 1 Division of Epidemiology, Department of Environmental and Public Health Sciences, University of Cincinnati College of Medicine, 160 Panzeca Way, Room 335, Cincinnati, OH 45267 USA; 2 Division of Asthma Research, Department of Pediatrics, Cincinnati Children’s Hospital Medical Center, University of Cincinnati, Cincinnati, OH USA

**Keywords:** Epigenetic clock, Epigenetic age acceleration, Mortality, Cardiovascular disease, Cancer

## Abstract

Epigenetic clocks have emerged as novel measures of biological aging and potential predictors of mortality. We examined all-cause, cardiovascular, and cancer mortality prediction by epigenetic age acceleration (EAA) estimated using different epigenetic clocks. Among 2105 participants to the 1999–2002 National Health and Nutrition Examination Survey aged ≥ 50 years old and followed for mortality through 2019, we calculated EAAs from the residuals of nine epigenetic clocks regressed on chronological age. We assessed the association of EAAs and pace of aging with mortality adjusting for covariates. During 17.5 years of median follow-up, 998 deaths occurred, including 272 from cardiovascular disease and 209 from cancer. Overall mortality was most significantly predicted by Grim EAA (*P* < 0.0001) followed by Hannum (*P* = 0.005), Pheno (*P* = 0.004), Horvath (*P* = 0.03), and Vidal-Bralo (*P* = 0.04) EAAs. Grim EAA predicted cardiovascular mortality (*P* < 0.0001), whereas Hannum (*P* = 0.006), Horvath (*P* = 0.009), and Grim (*P* = 0.01) EAAs predicted cancer mortality. Overall mortality prediction differed by race/ethnicity between non-Hispanic White and White participants for Horvath (*P*_interaction_ = 0.048), Hannum (*P*_interaction_ = 0.01), and Grim (*P*_interaction_ = 0.04) EAAs. Hannum prediction of cancer mortality also differed between the two races/ethnicities (*P*_interaction_ = 0.007). Despite being predictive in non-Hispanic White participants, Horvath (*P* = 0.75), Hannum (*P* = 0.84), and Grim (*P* = 0.10) EAAs failed to predict overall mortality in Hispanic participants, and Hannum EAA was not associated with cancer mortality in Hispanic participants (*P* = 0.18). In a US representative sample, Horvath, Hannum, SkinBlood, Pheno, Vidal-Bralo, and Grim EAAs as well as pace of aging predict mortality. Howbeit, Horvath, Hannum, and Grim EAAs were less predictive in Hispanic participants.

## Introduction

Epigenetic modifications or the alterations in gene activity without changes in the DNA sequence potentially transmitted to the organism’s offspring through a process called transgenerational epigenetic inheritance, are a well-known phenomenon associated with aging [1]. Epigenetic processes include DNA methylation (DNAm), histone modifications, and the non-coding RNAs that alter the binding of proteins to DNA and induce or repress gene transcription [2]. Among epigenetic markers, DNAm is the most stable and quantifiable; it is the selective addition of a methyl group to cytosine within cytosine-phosphate-guanine (CpG) dinucleotide sites to form 5-methylcytosine (5mC) [3]. As early as 1973, Vanyushin et al. reported an inverse relationship between aging and 5-methylcytosine in DNA from rats’ tissues [4]. These findings were subsequently replicated in various species including humans, leading to the genomic hypomethylation hypothesis of aging, which suggested that reduced 5mC and lower global DNAm occur with aging and may cause a relaxation and abnormal gene expression [2]. Later research showed that global 5mC does not consistently vary with age; however, DNAm alterations at specific sites of the genome occur with aging [2]. Such epigenetic alterations are considered as a silent indicator for aging and age‐associated health risks [5].

In recent years, epigenetic clocks built from sets of CpGs using mathematical algorithms have emerged as promising measures of biological aging and may improve mortality prediction [6]. The first generation of epigenetic clocks was developed by Horvarth, Hannum, and Weidner to correlate with chronological age [7–9]. The Horvath clock was developed with DNAm data from various human tissues based on 353 CpGs to predict chronological age with 3.6 years mean deviance [9]. The Hannum and Weidner clocks were based on 71 CpGs and 3 CpGs, respectively and were developed with DNAm data from whole blood [7, 8]. Zhang et al. attempted to improve the performance of these clocks in predicting chronological age by increasing the training sample size, which resulted in the selection of 514 CpGs from whole blood and saliva [10]. The Vidal-Bralo clock was another first-generation clock; it included eight whole blood CpGs weighted for their methylation values to predict chronological age [11]. Second-generation clocks such as PhenoAge (513 CpGs) and GrimAge (1030 CpGs) were built with from whole blood DNAm to powerfully predict not only mortality, but also morbidity [12, 13]. The SkinBloodAge clock was developed using 391 CpGs from skin and whole blood to improve DNAm age estimation of previous clocks in fibroblasts and other cell types used in ex vivo studies [14]. Lin et al. also produced a clock with 99 whole blood CpGs that correlated with chronological age and cancer [15]. Third-generation clocks such as DunedinPoAm estimate the pace of cellular aging using biomarkers of organ system dysfunction [16].

Although there are several methods developed to estimate epigenetic clocks in specific conditions, few studies have assessed the ability of epigenetic age acceleration (EAA) to predict all-cause and cause-specific mortality in general populations [17–19]. Therefore, we aimed to determine the association of EAA estimated using epigenetic clocks of first, second, and third generation with overall as well as cardiovascular and cancer mortality in a sample representative of the US population aged 50 years or older.

## Methods

### Study participants

We used data from the National Health and Nutrition Examination Survey (NHANES) conducted from 1999 to 2002 that included data on epigenetic age. The NHANES is a survey conducted by the National Center for Health Statistics (NCHS) of the Centers for Disease Control and Prevention (CDC) to evaluate the health and nutritional status of US non‐institutionalized civilian population [20]. It uses a complex multistage sampling design to derive a sample that is nationally representative [20]. Written informed consent was obtained from all the study participants, and the study design as well as the study protocols were approved by the CDC and NCHS institutional review boards [20].

Among the 2532 NHANES 1999–2002 participants who had data on epigenetic age, we excluded participants with missing data on pack-years of cigarette smoking (*N* = 138), body mass index (BMI) (*N* = 53), poverty income ratio (BMI) (*N* = 183). The final sample used for analysis was 2105 participants.

### Epigenetic clocks

DNA was extracted from whole blood and stored at − 80 °C until DNA methylation analysis at the Duke University Molecular Physiology Institute. The bisulfite conversion of DNA was performed using a Zymo EZ DNA methylation kit (Zymo Research, Irvine, California) applying conditions for the Illumina Infinium Methylation assay. DNAm results were produced on the Illumina Infinium MethylationEPIC BeadChi (Illumina, San Diego, California). The data was processed, normalized, and used to derive epigenetic biomarkers for the Horvath, Hannum, SkinBlood, Pheno, Zhang, Lin, Weidner, Vidal-Bralo, and GrimAge2 clocks, which were all the epigenetic clocks publicly available in NHANES. Pace of aging was estimated from the DunedinPoAm. Detailed descriptions of the laboratory procedures, DNAm analysis, and quality control are provided at https://wwwn.cdc.gov/nchs/data/nhanes/dnam/NHANES%20DNAm%20Epigenetic%20Biomarkers%20Data%20Documentation.pdf.

### Overall, cardiovascular, and cancer mortality

The NCHS matched NHANES participants to their National Death Index (NDI) records and used the death certificates for confirmation. Overall mortality was defined as deaths from any cause, excluding mortality from accidents. The specific causes of mortality in our analysis was defined using a standardized list of 113 causes according to the Tenth Revision of the International Classification of Diseases (ICD-10) and included mortality from cardiovascular disease (diseases of the heart) (ICD-10 codes I00-I09, I11, I13, I20-I51) and cancer (ICD-10 codes C00-C97) [21]. The small number of deaths from other causes precluded any further stratification.

### Covariates

Baseline information on participants chronological age, sex, race/ethnicity, annual household income, cigarette smoking, physical activity, and comorbidities were collected using questionnaires. Using guidelines household income adjusted for family size, year of survey, and state, the NCHS estimated PIR [22]. Participants were asked about moderate to vigorous physical activity in the past 30 days [23]. BMI was calculated as measured weight in kilograms over height in meter squared [23]. We defined diabetes as self-reported treatment by oral antidiabetic drug or insulin, fasting plasma glucose ≥ 126 mg/dl or hemoglobin A_1_C ≥ 6.5% [23]. We defined hypertension as self-reported use of antihypertension drugs, mean systolic blood pressure ≥ 140 mm Hg or diastolic blood pressure ≥ 90 mm (of up to four measurements on two separate occasions) [23]. Complete blood count measurements were done with the Beckman Coulter method, and white blood cell (WBC) differential was performed using VCS technology [24].

### Statistical analysis

We inspected the intercorrelation between the different epigenetic clocks as well as their correlation with chronological age and pace of aging using Pearson correlations. We performed descriptive analyses to report the distribution of study participants characteristics, and we estimated the mean and corresponding standard error (SE) of epigenetic age, epigenetic age acceleration (EAA), and pace of aging. Epigenetic age acceleration (EAA) was calculated for each of the epigenetic clocks by computing the residuals from the regression of epigenetic age on chronological age [18]. To examine the association of EAA using the different epigenetic clocks and pace of aging with overall and cause-specific mortality, we performed Cox proportional hazards regression and estimated the hazard ratio (HR) with corresponding 95% confidence interval (CI). We tested for the proportionality assumption by including interaction terms for each independent variable with follow-up time, and none of the interactions was significant, which indicated that no violation was identified [21]. The models were adjusted for chronological age, PIR, pack-years of cigarette smoking, and WBC composition used as continuous variables; and sex, race/ethnicity, smoking status, BMI, physical activity, asthma, diabetes, and hypertension used as categorical variables. We reported HRs for 5-year increases in EAA and 10% increases in the pace of aging for meaningful interpretation and report of the results. To examine whether mortality prediction by EAA differed by race/ethnicity, we tested effect modification using an interaction term and performed stratified analysis by modifiers found to be significant. We accounted for the NHANES complex survey design and the sampling weights, so that our results were nationally representative. All analyses were performed in SAS (Version 9.4), and two-sided *P* values < 0.05 were considered significant.

## Results

### Descriptive results

The study population consisted of 2105 participants followed for up to 20.7 years (median 17.5 years [interquartile range 10.4, 18.7]) during which 998 deaths occurred, including 272 from cardiovascular disease and 209 from cancer. The baseline characteristics of study participants are reported in Table 1. In summary, the mean (SE) age of participants was 63.96 (0.34); the majority (54.6%) were female; 79.4% were non-Hispanic White versus 8.3% non-Hispanic Black and 9.1% Mexican American. About 29.9% had a PIR < 1.85; frequency of current or past smoking was 54.0%; 70.8% were overweight or obese; and 53.5% reported moderate or vigorous physical activity in the past 30 days. The prevalence of comorbidities was 5.2% for asthma, 12.1% for asthma, and 56.0% for hypertension.

**Table 1:** Baseline characteristics of study participants, NHANES 1999–2002 (*N* = 2105)

Characteristics	Estimate
Chronological age (years), mean (SE)	63.96 (0.34)
Epigenetic age (years), mean (SE)	
Horvath	65.50 (0.30)
Hannum	65.16 (0.29)
SkinBlood	62.16 (0.29)
Pheno	53.67 (0.37)
Zhang	66.11 (0.12)
Lin	55.66 (0.43)
Weidner	53.56 (0.44)
Vidal-Bralo	60.09 (0.25)
Grim	69.98 (0.34)
Female sex, %	54.6
Race/ethnicity, %	
Non-Hispanic White	79.4
Non-Hispanic Black	8.3
Mexican American	9.1
Hispanic and Other	3.3
PIR < 1.85, %	29.9
Smoking, %	
Never	46.0
Past smoker	39.1
Current smoker	14.9
BMI, %	
Underweight (< 18.5 kg/m^2^)	0.9
Normal (18.5 – 24.9 kg/m^2^)	28.3
Overweight (25.0 – 29.9 kg/m^2^)	37.5
Obese (≥ 30.0 kg/m^2^)	33.3
Physical activity in the past 30 days, %	53.9
Current asthma, %	5.2
Diabetes, %	12.1
Hypertension, %	56.0
Epigenetic age – chronological age, mean (SE)	
Horvath	1.54 (0.23)
Hannum	1.21 (0.20)
SkinBlood	−1.44 (0.16)
Pheno	−10.28 (0.22)
Zhang	2.15 (0.25)
Lin	−8.29 (0.26)
Weidner	−10.40 (0.39)
Vidal-Bralo	−3.87 (0.22)
Grim	6.03 (0.24)
DunedinPoAm	1.10 (0.005)

*SE*, standard error; *PIR*, poverty income ratio; *BMI*, body mass index; *EAA*, epigenetic age acceleration

On average, epigenetic age was accelerated with the Horvath (mean [SE] 1.55 [0.23]), Hannum (mean [SE] 1.20 [0.20]), Zhang (mean [SE] 2.15 [0.25]), and Grim (mean [SE] 6.03 [0.23]) clocks. However, it decelerated with the SkinBlood (mean [SE] − 1.44 [0.16]), PhenoAge (mean [SE]: − 10.28 [0.22]), Lin (mean [SE] − 8.29 [0.26]), Weidner (mean [SE] − 10.40 [0.39]), and Vidal-Bralo (mean [SE] − 3.87 [0.22]) epigenetic clocks (Table 1).

### Correlation with chronological age and between epigenetic clocks

**Fig. 1:**
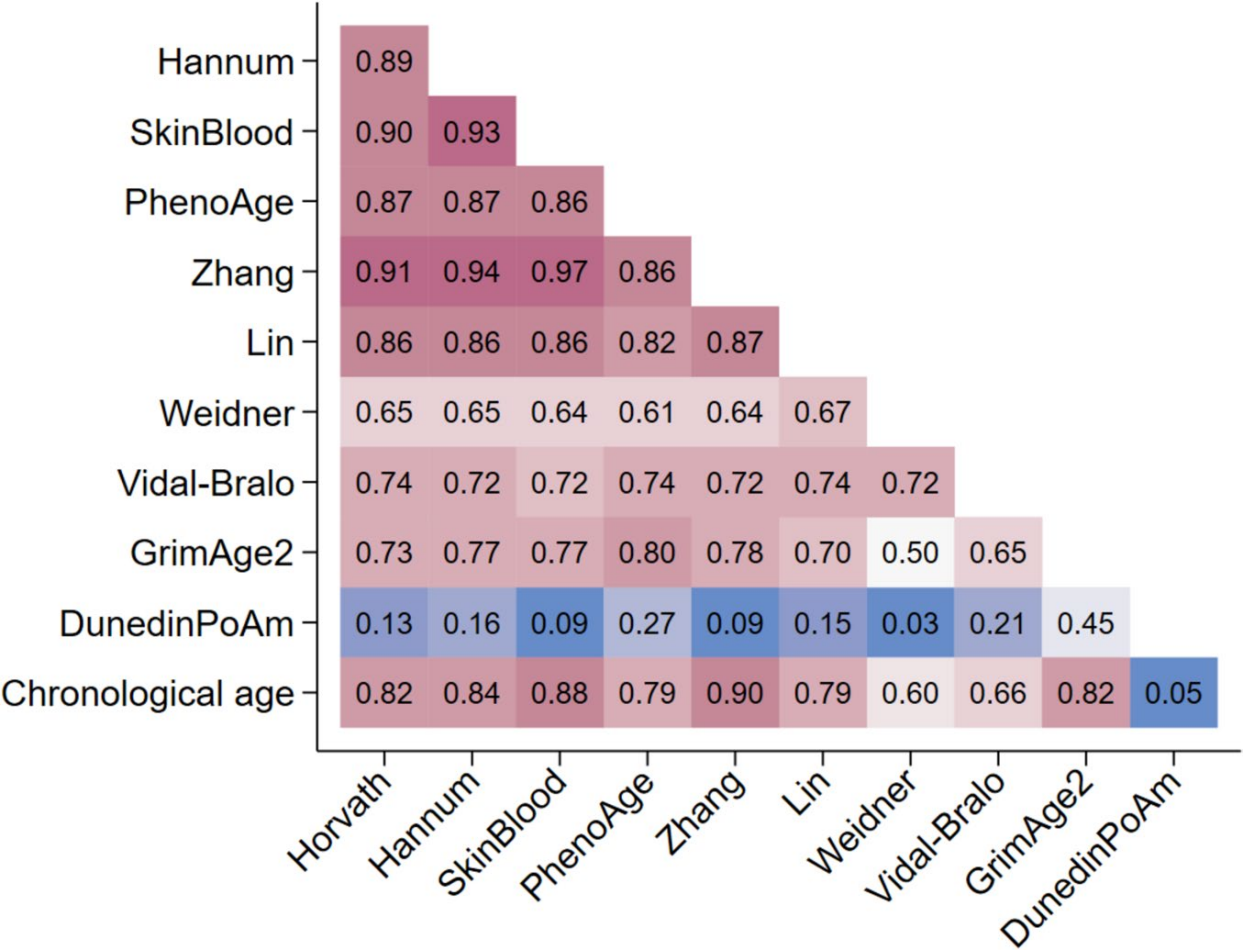
Intercorrelation between epigenetic clocks and correlation with pace of aging and chronological age

The strongest correlation between epigenetic clocks and chronological age was observed for Zhang (*r* = 0.90), followed in decreasing order by SkinBlood (*r* = 0.88), Hannum (*r* = 0.88), Horvath (*r* = 0.82), GimAge2 (*r* = 0.82), PhenoAge (*r* = 0.79), Lin, Vidal-Bralo (*r* = 0.66), and Weidner (*r* = 0.60) clocks. Very strong intercorrelations (*r* ≥ 0.80) were observed between the Horvath, Hannum, SkinBlood, PhenoAge, and Zhang clocks. The correlation coefficients of GrimAge2 with the other clocks ranged from 0.65 to 0.80; those of Vidal-Bralo with the other clocks were between 0.65 and 0.47, and those of Weidner with the other clocks were from 0.50 to 0.72 (Fig. 1).

### EAA and mortality

In Cox proportional hazards regression adjusted for covariates and by order of decreasing significance, higher overall mortality was predicted by EAA calculated with the Grim (*P* < 0.0001; HR 1.51, 95% CI 1.33, 1.70), Pheno (*P* = 0.004; HR 1.12, 95% CI 1.04, 1.20), Hannum (*P* = 0.005; HR 1.14, 95% CI 1.04, 1.24), Horvath (*P* = 0.03; HR 1.11, 95% CI 1.01, 1.22), and Vidal-Bralo (*P* = 0.04; HR 1.10, 95% CI 1.00, 1.21) clocks. In cause-specific mortality, only Grim EAA was predictive of cardiovascular mortality (*P* < 0001; HR 1.61, 95% CI 1.32, 1.96) whereas Hannum (*P* = 0.006; HR 1.27, 95% CI 1.08, 1.49), Horvath (*P* = 0.009; HR 1.22, 95% CI 1.06, 1.42), and Grim (*P* = 0.01; HR 1.46, 95% CI 1.08, 1.96) EAAs predicted cancer mortality. The DunedinPoAm pace of aging was associated with overall (*P* = 0.0008; HR 1.21, 95% CI 1.09, 1.35) and cardiovascular mortality (*P* = 0.04; HR 1.24, 95% CI 1.01, 1.54) (Table 2).

**Table 2:** Epigenetic age acceleration (EAA) and overall, cardiovascular or cancer mortality, NHANES 1999–2002

EAA	Overall mortality (998 deaths)		Cardiovascular mortality (272 deaths)		Cancer mortality (209 deaths)	
	HR (95% CI)	*P* value	HR (95% CI)	*P* value	HR (95% CI)	*P* value
Horvath	**1.11 (1.01, 1.22)**	**0.03**	1.06 (0.94, 1.20)	0.32	**1.22 (1.06, 1.42)**	**0.009**
Hannum	**1.14 (1.04, 1.24)**	**0.005**	1.09 (0.92, 1.29)	0.30	**1.27 (1.08, 1.49)**	**0.006**
SkinBlood	1.03 (0.94, 1.12)	0.52	1.19 (0.95, 1.48)	0.12	0.98 (0.86, 1.11)	0.71
Pheno	**1.12 (1.04, 1.20)**	**0.004**	1.01 (0.91, 1.13)	0.79	1.13 (0.97, 1.33)	0.12
Zhang	1.19 (0.92, 1.53)	0.18	1.28 (0.82, 2.01)	0.27	1.07 (0.72, 1.60)	0.71
Lin	1.03 (0.97, 1.09)	0.29	1.03 (0.95, 1.11)	0.42	1.04 (0.93, 1.16)	0.48
Weidner	1.02 (0.98, 1.06)	0.28	1.01 (0.93, 1.09)	0.82	1.04 (0.95, 1.14)	0.42
Vidal-Bralo	**1.10 (1.00, 1.21)**	**0.04**	1.05 (0.92, 1.21)	0.45	1.17 (0.98, 1.40)	0.08
Grim	**1.51 (1.33, 1.70)**	** < 0.0001**	**1.61 (1.32, 1.96)**	** < 0.0001**	**1.46 (1.08, 1.96)**	**0.01**
DunedinPoAm	**1.21 (1.09, 1.35)**	**0.0008**	**1.24 (1.01, 1.54)**	**0.04**	1.03 (0.75, 1.40)	0.86

*HR*, hazard ratio; *CI*, confidence interval; *EAA*, epigenetic age acceleration

Hazard ratios calculated using Cox proportional hazards regression. Models adjusted for chronological age, poverty income ratio, smoking pack-years, and white blood cell composition used as continuous variables; and gender, race/ethnicity, smoking status, physical activity, body mass index, asthma, diabetes, and hypertension used as categorical variables. HR reported per 5-year increases in EAA and per 10% increase in DunedinPoAm pace of aging. **Bold** and grey shade indicate significant association between EAA and mortality outcome

### Effect modification by race/ethnicity on EAA and mortality

There were significant differences by race/ethnicity (NH White vs. Hispanic) in the associations of Horvath EAA with overall mortality (*P*_interaction_ = 0.048), of Hannum EAA with overall (*P*_interaction_ = 0.01) and cancer mortality (*P*_interaction_ = 0.007), and of Grim EAA with overall mortality (*P*_interaction_ = 0.04). Overall mortality was predicted by Horvath (HR 1.14, 95% CI 1.02, 1.27), Hannum (HR 1.15, 95% CI 1.04, 1.28), and Grim (HR 1.55, 95% CI 1.33, 1.82) EAAs in NH White but not Hispanic participants. Likewise, Hannum predicted cancer mortality in NH White but not Hispanic participants (HR 1.36, 95% CI 1.14, 1.62) (Tables 3 and 4).

**Table 3:** *P* value for effect modification by race/ethnicity on EAA and overall, cardiovascular, and cancer mortality, NHANES 1999–2002

EAA and subgroups	All-cause mortality	Cardiovascular mortality	Cancer mortality
Horvath			
NH Black vs. NH White	0.93	0.72	0.45
Hispanic vs. NH White	**0.048**	0.15	0.16
Hannum			
NH Black vs. NH White	0.43	0.95	0.91
Hispanic vs. NH White	**0.01**	0.67	**0.007**
SkinBlood			
NH Black vs. NH White	0.81	0.12	0.57
Hispanic vs. NH White	0.11	0.67	0.26
Pheno			
NH Black vs. NH White	0.89	0.25	0.50
Hispanic vs. NH White	0.20	0.33	0.44
Zhang			
NH Black vs. NH White	0.52	0.22	0.94
Hispanic vs. NH White	0.38	0.92	0.25
Lin			
NH Black vs. NH White	0.19	0.64	0.86
Hispanic vs. NH White	0.55	0.46	0.15
Weidner			
NH Black vs. NH White	0.18	0.38	0.84
Hispanic vs. NH White	0.80	0.13	0.94
Vidal-Bralo			
NH Black vs. NH White	0.78	0.64	0.95
Hispanic vs. NH White	0.57	0.17	0.91
Grim			
NH Black vs. NH White	0.83	0.17	0.53
Hispanic vs. NH White	**0.04**	0.27	0.25
DunedinPoAm			
NH Black vs. NH White	0.94	0.22	0.19
Hispanic vs. NH White	0.25	0.82	0.82

*HR*, hazard ratio, *CI*, confidence interval, *EAA*, epigenetic age acceleration, *BMI*, body mass index, *NH*, non-Hispanic

Hazard ratios calculated using Cox proportional hazards regression. Models adjusted for chronological age, poverty income ratio, body mass index, pack-years of cigarette smoking, and white blood cell composition used as continuous variables; and gender, race/ethnicity, smoking status, physical activity, asthma, diabetes, and hypertension used as categorical variables. **Bold** and grey shade indicate significant association between EAA and mortality outcome

**Table 4:** Stratified analysis by effect modifiers of the association between EAAs and mortality, NHANES 1999–2002

EAA and subgroups	All-cause mortality			Cancer mortality		
	HR (95% CI)	*P*	*P*_interaction_	HR (95% CI)	*P*	*P*_interaction_
Horvath						
By race/ethnicity			**0.048**			
NH White	**1.14 (1.02, 1.27)**	**0.02**		Not applicable		
Hispanic	0.98 (0.84, 1.14)	0.75		Not applicable		
Hannum						
By race/ethnicity			**0.01**			**0.007**
NH White	**1.15 (1.04, 1.28)**	**0.009**		**1.36 (1.14, 1.62)**	**0.001**	
Hispanic	0.99 (0.90, 1.09)	0.84		0.85 (0.67, 1.08)	0.18	
Grim						
By race/ethnicity			**0.04**			
NH White	**1.55 (1.33, 1.82)**	** < 0.0001**		Not applicable		
Hispanic	1.29 (0.95, 1.74)	0.10		Not applicable		

*HR*, hazard ratio; *CI*, confidence interval; *EAA*, epigenetic age acceleration; *BMI*, body mass index; *NH*. non-Hispanic

Hazard ratios calculated using Cox proportional hazards regression. Models adjusted for chronological age, poverty income ratio, body mass index, pack-years of cigarette smoking, and white blood cell composition used as continuous variables; and gender, race/ethnicity, smoking status, physical activity, asthma, diabetes, hypertension used as categorical variables. **Bold** and gray shade indicate significant association between EAA and mortality outcome

## Discussion

In a sample representation of the US population aged 50 years or older, Horvath, Hannum, Pheno, Vidal-Bralo, and Grim EAAs predicted overall mortality. Cardiovascular mortality was predicted by Grim EAA, and cancer mortality was predicted by Horvath, Hannum, and Grimm EAAs. However, overall mortality was predicted by Horvath, Hannum, and Grim EAAs in non-Hispanic White but not in Hispanic participants, and cancer mortality was predicted by Hannum in non-Hispanic White but not Hispanic participants. Pace of aging was predictive of overall and cardiovascular but not cancer mortality in all participants, without effect modification by race/ethnicity.

This is the first US representative analysis to examine EAA’s association with mortality from specific causes such as cardiovascular disease or cancer and the first to evaluate mortality prediction by EAA developed from the Zhang, Lin, Weidner, and Vidal-Bralo epigenetic clocks. Among 3581 participants to the US Health and Retirement Study (HRS) aged 50 years or older, Hannum, Pheno, and Grim EAAs and the DunedinPACE rate of aging, but not Horvath EAA, predicted 4-year all-cause mortality after adjusting for sociodemographic characteristics, health behavior, and cell types [18]. Likewise in the US Women’s Health Initiative (WHI) study, Horvath, Hannum, Pheno, and Grim EAAs were associated with lower odds of healthy longevity (survival to age 90 with intact mobility) among 1813 older women [17]. In Germany, Horvath EAA was associated with all-cause, cancer, and cardiovascular mortality, whereas the Hannum EAA was only associated with all-cause mortality in 1864 participants 50 to 75 years old adjusted for chronological age, sex, batch effects, and leukocyte distribution [19]. After further adjustment for smoking, BMI, and comorbidities, only the Horvath EAA was associated with all-cause and cancer mortality [19]. In the Scottish Family Health Study, Grim EAA, but not Horvath, Hannum nor Pheno EAAs, predicted all-cause mortality in 9537 followed for 13 years [25]. In summary, our results confirmed previous reports of all-cause mortality prediction by Horvath, Hannum, Pheno, and Grim EAAs as well as pace of aging. They also report novel mortality prediction by Vidal-Bralo EAA.

In cause-specific mortality, we observed that only Grim EAA predicted cardiovascular mortality. Also, Hillary et al. reported that GrimAge, but not Horvath, Hannum, nor Pheno epigenetic clocks, predicted ischemic heart disease in the Scottish Family Health Study [25]. Similar results were found in African American participants to the Genetic Epidemiology Network of Arteriopathy (GENOA) study where Grim, but not Pheno EAA was associated with cardiovascular disease incidence, independent of traditional risk factors [26]. The association of Horvath and Hannum EAAs with cardiovascular outcomes has been inconsistent. In the Atherosclerosis Risk in Communities (ARIC) Study, these EAA measures were associated with cardiovascular events and mortality among 2543 African Americans over 21 years, after adjusting for chronological age, sex, and cell types [27]. However, the association with cardiovascular mortality became non-significant when smoking, physical activity, BMI, diabetes, and hypertension (all of which were included in our analysis) were not adjusted for [27]. In summary, our results confirmed previous reports of Grim EAA association with cardiovascular mortality.

Consistent with our results on cancer mortality related to Horvath, Hannum, and Grim EAAs, other reported epidemiology studies have reported associations of EAA with incident colorectal cancer, breast cancer in postmenopausal women, and lung cancer [28–31]. Also similar to our results, pace of aging was shown to be associated with overall and cardiovascular mortality and was suggested to mediate the association of life’s essential 8 (diet, physical activity, nicotine exposure, sleep, BMI, blood lipids, glucose, and blood pressure) with these outcomes [32]. The underlying mechanisms for the associations of EAAs with overall and cause-specific mortality are unclear and beyond the scope of the present analysis aimed at identifying the EAA algorithms that best predict these outcomes. EAA may capture long-term exposures associated with adverse health effects and aging as well hormonal, inflammatory, and metabolic processes that play a role in disease [30]. The inconsistent results observed with EAA estimated from various epigenetic clocks may be explained by differences in training data sets, CpGs included in the prediction, the clinical inputs, and statistical methods [30]. In summary, despite the inconsistent literature, our findings confirmed reports of Horvath, Hannum, and Grim EAAs being predictive of cancer mortality.

We found that overall mortality prediction by Horvath and Hannum differed between non-Hispanic White and Hispanic participants, but the training data for the development of these clocks included both racial/ethnic groups, and there were strong correlations between the epigenetic clocks and chronological age [33]. As a possible explanation, Breeze et al. reported that in the Internation Human Epigenome Consortium (IHEC), which includes datasets on gene expression across several tissues and cell types, 87.1% of publicly available experiments were performed in European ancestry participants versus 1.7% in African ancestry and 1.2% in Hispanic ancestry [34]. They also found that the breadth of epigenomic essays was more extensive in European than non-European ancestries [34]. The effect of ancestry-related genetic variants on epigenetic modifications is also unknown. Yet, we found difference only in non-Hispanic White versus Hispanic participants but not between non-Hispanic White and Black participants.

Our analysis had limitations. EAAs were estimated in DNAm extracted from whole blood at a single time point, and how they may change over time is unclear. We did not have information on the incidence of cardiovascular disease or cancer and on mortality from specific types of cancer. As for any observational studies, residual confounding cannot be completely ruled-out; however, attempts were made to reduce it by adjusting for a wide range of relevant covariates and potential confounders. Nonetheless, this analysis has major strengths. It includes a sample representative of the US older adult population which increases the generalizability of our results. DNA methylation and epigenetic clocks were estimated with rigorous methods and QC using laboratory selected by CDC.

In conclusion, in a sample representative of the US population aged 50 years or older, Horvath, Hannum, Pheno, Vidal-Bralo, and Grim EAAs all predicted overall mortality; Grim EAA predicted cardiovascular mortality; and Horvath, Hannum, as well as Grim EAAs predicted cancer mortality. However, EAA calculated using Horvath, Hannum, or Grim clocks seemed less predictive in Hispanic participants. The pace of aging was predictive of overall and cardiovascular but not cancer mortality, regardless of race/ethnicity.

## Data Availability

All the data sets used for this article's analysis are publicly available and can be dowloaded at https://www.cdc.gov/nchs/nhanes/index.html.
